# Relationships Between Rearing Enrichments, Range Use, and an Environmental Stressor for Free-Range Laying Hen Welfare

**DOI:** 10.3389/fvets.2020.00480

**Published:** 2020-08-14

**Authors:** Md Saiful Bari, Jeff A. Downing, Tim R. Dyall, Caroline Lee, Dana L. M. Campbell

**Affiliations:** ^1^School of Environmental and Rural Science, University of New England, Armidale, NSW, Australia; ^2^Agriculture and Food, Commonwealth Scientific and Industrial Research Organisation (CSIRO), Armidale, NSW, Australia; ^3^Department of Dairy and Poultry Science, Chattogram Veterinary and Animal Sciences University, Chattogram, Bangladesh; ^4^Faculty of Veterinary Science, School of Life and Environmental Science, University of Sydney, Sydney, NSW, Australia

**Keywords:** novel objects, perching structures, range access, plumage coverage, corticosterone, RFID, behavior, egg quality

## Abstract

Enrichments during pullet rearing may improve adaptation and welfare of hens as they move from indoor rearing to a free-range system. Individual variation in outdoor ranging may also affect welfare. This study assessed the effects of rearing enrichments and an imposed environmental stressor on hen welfare and egg quality along with the association of welfare with ranging. Hy-Line Brown® chicks (*n* = 1,386) were reared indoors until 16 weeks with 3 enrichment treatments including a “control” group with standard floor litter, a “novelty” group that received novel objects that were changed weekly, and a “structural” group with H-shaped perching structures. Pullets were then moved to a free-range system with three replicates of each rearing treatment. Daily ranging was individually tracked from 25 to 64 weeks via radiofrequency identification technology. Individual hen welfare assessments were performed at 25, 33, 43, 56, and 64 weeks and correlated with ranging time prior to these dates. At 44 weeks, the range area was reduced by 80% for 11 days to induce stress. Changes in ranging behavior, albumen corticosterone concentrations and egg quality were evaluated. GLMMs showed significant interactions between hen age and rearing treatment for live weight, number of comb wounds, plumage coverage, and toenail length (all *P* ≤ 0.003), with the enriched hens showing more consistent live weight at the later ages, fewer comb wounds at 33 weeks, and better plumage coverage at the later ages, whereas the structural hens had shorter toenails as age increased. Plumage coverage showed a positive relationship with range use across most age points (*P* < 0.0001). Hens reduced ranging time following the imposed stressor but increased their number of visits with the lowest increase by the structural hens (*P* = 0.03). Significant interactions between rearing treatment and stressor for albumen corticosterone concentrations showed the structural hens decreased concentrations immediately post-stress, but the control and novelty groups increased (*P* < 0.006). The stressor increased or decreased values of most egg quality parameters across all rearing groups (all *P* ≤ 0.02). Overall, provision of rearing enrichments and greater range use may have positive impacts on hen welfare.

## Introduction

Animal welfare concerns are prevalent within the consumer community with apprehensions regarding the housing and management of livestock and desires for improvements that result in greater well-being for production animals (1–4). Specifically, in the poultry sector, free-range egg production is increasing as consumers perceive these hens produce tastier, healthier (5, 6), and more welfare-friendly (2, 7) eggs. Consumers believe that fresh air and outdoor access for birds in the free-range system improve hen welfare (3). However, laying hens demonstrate marked individual dissimilarities in range use when provided with outdoor access, which may result in individual differences in welfare (8). Outdoor access and time spent ranging, a higher proportion of hens ranging, or distance of ranging by free-range hens may result in some welfare benefits to the birds. This may include improved plumage coverage (9–11), reduced incidences of severe feather pecking (12), reduced footpad lesions (10) and reduced toenail length (13, 14). However, Larsen et al. (15) found limited association between frequency of range access and comb color, beak, footpad, and plumage condition although hens that ranged farther from the shed did have darker combs and less beak damage. In a sample of hens from the larger flock used in the current study, high outdoor access resulted in improved plumage coverage, a reduced number of pecking comb wounds, and reduced toenail length toward the end of the production cycle (16). There was also a negative relationship between ranging and body weight (specifically fat and muscle) in hens that spent the longest time outside (16). However, other research has shown limited relationships between body weight and range use (15, 17). Thus, research specifically examining the longitudinal relationship between individual range use patterns and welfare parameters will provide further insight.

In Australia, pullets reared for free-range systems cannot go outdoors due to health risks and the sheds not being designed accordingly, whereas adults have range access. This dissimilarity between rearing and adult housing might affect their adaptation to the range and subsequent welfare as similar rearing and laying housing environments are recommended for hens (18) to achieve better health and welfare outcomes. Enrichments during pullet rearing might contribute to overcoming the constraint of indoor rearing for free-range hens. For example, providing periodically altered novel objects may increase the adaptation to unpredictable environments as could be experienced during outdoor ranging as adults (19), or placing perching structures in the pullet shed may improve spatial navigation (20). More enriched pullet housing might also reduce stress and improve adaptability (21). In a previous study carried out at the same facility as the current study, chicks were provided with a variety of enrichments for the first 3 weeks of life compared with standard floor litter (19). When environmental stressors were applied, the enriched hens showed lower albumen corticosterone responses compared with the non-enriched hens indicating a reduced stress response (19).

Environmental stressors can have negative impacts on the production and welfare of laying hens. Common stressors include high stocking density, changes in management practice, changes in the social environment, or changes in resource access and can result in physiological welfare impacts such as increased stress hormones and/or changes in behavioral patterns (19, 22–24) although not in all cases (25). The impacts of these stressors may also manifest as changes in egg quality where varying parameters have been shown to be impacted by dietary corticosterone (26) or environmental stressors such as temperature and infection (27). Other environmental causes of acute or chronic stress in laying hens may result in changes in their egg quality.

In this context, the study was performed to assess the effect of rearing enrichments on, and associations of individual ranging patterns with, welfare parameters of free-range laying hens across the flock cycle along with hens' adaptability to an environmental stressor. We predicted better welfare in high outdoor ranging birds over the indoor hens along with better welfare and adaptability of the hens enriched during rearing than the control hens.

## Materials and Methods

### Ethical Statement

The research procedures were approved by the Animal Ethics Committee of the University of New England, Australia (AEC17-092).

### Animals

The study was conducted at the Rob Cumming Poultry Innovation Centre (indoors) and Laureldale poultry facility (free-range) of the University of New England, Armidale, NSW, Australia, using a total of 1386 Hy-Line® Brown layers. Surplus chicks were delivered in error and thus a total of 1,700 chicks were reared, but only 1,386 were transferred to the free-range facility. Surplus pullets that were of comparatively higher/lower body weight at 15 weeks of age and other randomly selected pullets from each pen were rehomed. A subsample of these hens at the end of the production cycle was reported on in Bari et al. (16) with similar data collection methods applied as described in the current study. The chicks and pullets were reared indoors within nine pens (6.2 m L × 3.2 m W) across three separate rooms up to 16 weeks of age, before being moved to the free-range facility and housed in nine pens within a single shed. The chicks and pullets were exposed to three enrichment treatments including a control group with a standard floor litter of rice hulls and no extra materials, a novelty group with different objects such as balls, bottles, bricks, brooms, brushes, buckets, containers, pet toys, and plastic pipes, that were added and changed weekly, and a structural group with four custom-designed H-shaped perching structures (L, W, H = 0.60 m) with two solid panels and one open-framed side. To visually isolate birds of each treatment group, shadecloth was hung on the wire pen dividers with each room having one replicate of each treatment (*n* = 3 replicates/treatment). The birds were provided *ad libitum* commercial mash feed placed in manual round feeders along with *ad libitum* water access from automatic nipple drinkers. The pullet density was approximately 15 kg/m^2^ (~9 birds/m^2^) (average 174–190 pullets/pen) at 16 weeks of age. All of the resources were provided to meet the requirements of the current Australian Model Code of Practice for the Welfare of Animals–Domestic Poultry (28). The management schedules of temperature and lighting were maintained as per the recommendations of the Hy-Line® Brown alternative management guidelines (29). However, as the pullets were intended to move outside in a free-range house as adults, artificial LED lighting was maintained at 100 lux. No cooling system was available, but mechanical ventilation with heating was provided as needed. Chicks were vaccinated as per regulatory requirements and standard recommendations including vaccination against Newcastle disease, Marek's disease, fowl pox, fowl cholera, egg drop syndrome, *Mycoplasma gallisepticum, Mycoplasma synoviae*, infectious bronchitis, infectious larngotracheitis, and avian encephalomyelitis. The chicks were also infra-red beak-trimmed at the hatchery.

The pullets at 16 weeks of age were re-housed in the Laureldale free-range facility across 9 indoor pens (*n* = 154 hens/pen; 3.6 m W × 4.8 m L) each with outdoor access via pop-holes. The pullets from different replicates of each treatment were socially re-mixed within their rearing treatments. Shadecloth visually isolated the indoor pens and outdoor range areas from each other. Rice hulls were used as floor litter, and a complete litter replacement was done at the mid-point of the flock cycle. Each pen was provided with nest boxes (two small and one large nest box), perches, two round hanging feeders, and water nipples to meet model code guidelines.

From 16 weeks of age onwards, the artificial LED lighting gradually increased to 16 h light and 8 h darkness by 30 weeks of age with an average light intensity of 10.0 (±0.84 SE) lux (Lutron Light Meter, LX-112850; Lutron Electronic Enterprise CO., Ltd, Taipei, Taiwan) for each pen as measured at birds' eye height from three pen locations (front, middle, back) when the pop-holes were closed. This light intensity (lux) was the highest that could be achieved with the shed lighting system. There was no automatic temperature and humidity control in the shed, but it was mechanically fan-ventilated.

For outdoor ranging, each pen was connected to an outdoor area (31 m L × 3.6 m W for each pen) that was accessed by the hens via two pop-hole openings (18 cm W × 36 cm H). The range area just after the pop-holes was 1.1 m of concrete path, then 1.6 m of river rock followed by a grassed area with no additional trees or shelter. The grassed area became bare dirt following both hen access and the winter season. Hens were provided access to the outdoor area from 25 weeks of age (May 2018) for most of the daytime via automatic opening and closing of the pop-holes. The pop-holes opened at 9:15 a.m. and closed after sunset daily. This equated to ~9 h of available ranging time daily across winter followed by ~11 h of available ranging time daily after daylight saving time began (October 2018 until February 2019).

### Radio-Frequency Identification System

Radio-frequency identification (RFID) systems (17) were placed within the pop-holes to track the hens' movement in and out of the pop-holes. The RFID systems were designed and supported by Microchips Australia Pty Ltd (Keysborough, VIC, Australia) with equipment developed and manufactured by Dorset Identification B.V. (Aalten, the Netherlands) using Trovan® technology. All hens were banded with microchips (Trovan® Unique ID 100 (FDX-A): operating frequency 128 kHz; Microchips Australia Pty Ltd) glued into adjustable leg bands (Roxan Developments Ltd, Selkirk, Scotland) with the system recording the date and time of each tagged bird passing through and in which direction (onto the range, or into the pen) with a precision of 0.024 s (maximum detection velocity 9.3 m/s). The individual ranging data were collected daily from 25 until 64 weeks of age (excluding some days when there was a technical malfunction or when there were experimental processes such as the weighing/scoring of hens).

### Extraction of Ranging Data

The individual-hen daily RFID data throughout the laying cycle from 25 to 64 weeks of age were collated into four daily-average sets to match the periodic welfare scoring of the hens (see section Individual Welfare Assessment); averages at 33 weeks (32 days up to 12 June 2018), 43 weeks (55 days up to 20 August 2018), 56 weeks (48 days up to 20 November 2018), and 64 weeks (70 days up to 30 January 2019) of age. The data were run through a custom-designed software program written in the “Delphi” language (Bryce Little, Agriculture and Food, CSIRO, St Lucia, QLD, Australia) that filtered out any unpaired or “false” readings that may occur if, for example, a hen sits inside the pop hole but does not complete a full transition onto the range or back into the pen. The software program then summarized the mean daily time (hours) outdoors per day for each of the hens across the different age periods. To assess the effect of the implemented stressor (shrinkage of ranging area, see section Environmental Stressor) on ranging behavior (time outside and also the number of visits to the range), the individual-hen data 10 days before the stressor was applied and 10 days during the stress were also compiled (ranging data during the stressor period were not included in the summaries for the welfare scoring age points).

### Individual Welfare Assessment

The welfare assessment of all hens was done individually at five age points including 25, 33, 43, 56, and 64 weeks of age. All hens were scored inside under bright working lights by the same trained scorer who was not blind to the rearing treatments but was unaware of individual hen ranging patterns. All the hens were weighed individually using electronic hanging scales (BAT1; VEIT Electronics, Moravany, Czech Republic). The external welfare parameters of feather loss at different body parts (neck, chest, back, wing, vent, tail) and footpad lesions were assessed using the scoring system described by Tauson et al. (30). In this scoring system, four scores were available for feather coverage where a score of 4 indicated minimal feather damage, and a score of 1 indicated no plumage, just bare skin. The back of the neck was scored separately from the front of the neck which was not included in the analyses as the majority of damage on the neck front was believed to have resulted from rubbing on the feeder rims rather than pecking damage. A maximum score of 24 could be obtained for feather condition across six body parts. Footpad lesions were scored as a four for a normal footpad with no lesions or dermatitis and a score of 1 for swollen, infected bumblefoot. The exact number of fresh or healing comb wounds was also counted (comb wounds were easily visible under the lights regardless of variation in comb color), and toenail length was measured in mm using a seamstress tape measure. Beaks were scored as 0, 1, or 2 indicating no, mild, or moderate damage, respectively. Beak damage was scored based on the evenness of the upper and lower mandibles including overgrowth or deformities which may have resulted from the day-old beak trimming procedure. The keels of each hen were scored by palpation as 1, 2, or 3 indicating normal (no damage), mild, or moderate damage, respectively. The birds were also examined for any other external signs of injury or illness such as a swollen abdomen, an enlarged crop or prolapse.

The mortality of hens was counted throughout the flock cycle. Hen mortality was recorded if a hen died, was euthanized, or rehomed if severely feather-pecked. A total of 28 hens were recorded throughout the cycle as the flock mortality of which nine were from the control group; nine, from the novelty group; and nine, from the structural group of rearing treatments.

### Environmental Stressor

The imposed environmental stressor for this trial was a reduction in available range area, similar to that applied in a previous study (19). The total outdoor area for each pen was reduced, using shadecloth, to ~20% of its original size (from 31 to 6 m L). The range area was reduced for 11 days from 44 to 45 weeks of age with egg measurements (see following sections on Egg Quality and Albumen Corticosterone) taken before the range shrinkage, the first days of shrinkage (immediate stress), and at the end of the stressor period (prolonged stress).

### Egg Quality

A total of 810 eggs were sampled at three time points with 270 sampled per time point (30/pen), collected randomly from all of the laying locations including small nests, large nests, and floors of the pens. The dirty eggs were excluded. The samples were first collected 4 days prior to stressor implementation as baseline samples; the same number of eggs was collected on Day 3 of shrinkage (immediate stress) and Day 10 of shrinkage as the prolonged stress samples. All the egg samples were individually tested for egg quality parameters including shell reflectivity, egg weight, breaking strength by quasi-static compression, shell deformation to breaking point, albumen height, Haugh Unit, yolk color score, shell weight, and shell thickness [Egg quality equipment; Technical Services and Supplies (TSS), Dunnington, York, UK]. Yolk color was measured digitally as a score based on color intensity corresponding to the DSM YolkFan (TSS equipment). Empty eggshells were then washed and left to dry for 24 h. The thickness of dried shells was measured at the eggshell equator in three places using a custom-made gauge based on a Mitutoyo Dial Comparator gauge (Model 2109-10). All the measurements of eggs were made on the day of collection (except eggshell thickness) by personnel blinded to the rearing treatment of the birds.

### Albumen Corticosterone

For the evaluation of concentrations of albumen corticosterone, a total of 50 eggs from each of the nine pens were sampled at three stages on the same days as the egg quality measurements; Day 4 prior to range shrinkage, and Days 3 and 10 following shrinkage. Eggs were collected from all laying locations but excessively dirty eggs were excluded. On the day of collection, the eggs were opened individually, the yolk was separated out and then the albumen was weighed and stored at −20°C until assessment using the validated radioimmunoassay reported by Downing and Bryden (31). All the egg corticosterone samples were analyzed blindly to the rearing treatments and implemented stressors.

### Data and Statistical Analyses

Statistical analyses were conducted in JMP® 14.0 (SAS Institute, Cary NC, USA) with α set at 0.05. The individual hen or sampled egg was the experimental unit. Data were transformed where needed but the raw values are presented in the tables and graphs. Non-significant interactions were removed from the final models. *Post-hoc* Student's *t*-tests were applied to the least-squares means where significant differences were present.

The welfare scoring data including the live weight, number of comb wounds, beak score, keel score, plumage score (total), and toenail length at different age points (25, 33, 43, 56, 64 weeks) throughout the laying cycle for individual hens from different rearing treatments were compiled (*n* = 6,876 data points/welfare parameter except for the beak score data which had *n* = 5,492 data points as beaks were not scored at the 25 week assessment date). The number of comb wounds and plumage score data were square-root-transformed, and the toenail length data were log_10_-transformed. For live weight, number of comb wounds, plumage score, and toenail length, general linear mixed models were fitted, with rearing treatment, age of hen, and their interaction as fixed effects and bird ID nested within pen nested with rearing treatment and pen nested within rearing treatment as random effects. The ordinal beak, keel and footpad scores were analyzed using an ordinal logistic regression with rearing treatments, age of hen and their interaction as fixed effects. The mean daily ranging time (h) of individual birds across all rearing treatments combined were correlated with the welfare parameters of live weight, beak score, keel score, number of comb wounds, plumage score (total), and toenail length of each bird using simple linear regressions separately for each age point. Ordinal logistic regressions were applied to the beak, keel, and footpad scores and ranging data. The *r*-values for each parameter were also calculated to display the direction of the relationship (*rho* values for the ordinal data).

The egg albumen corticosterone concentration data across the stressor period were compiled for the three different rearing treatments (*n* = 1,329 data points, 21 samples could not be accurately processed). A general linear mixed model was fitted, with rearing treatments, the stressor treatment, and their interaction as fixed effects and pen nested with rearing treatments as a random effect.

The average outdoor ranging time (h) per day and the average number of visits per day before the stress treatment and after applying stress were compiled for individual birds. The differences in ranging time (h) and number of visits per day were calculated per bird (*n* = 1,303 data points as ranging data were unavailable for hens that stayed inside). A positive difference number indicated a decrease in the number of visits/ranging time (h), and a negative difference number indicated an increase in the number of visits/ranging time (h) during the stressor period. General linear mixed models were fitted, with rearing treatment as a fixed effect and bird ID nested within pen nested with rearing treatment as a random effect.

Egg quality parameters measured from hens prior to stressing (baseline), immediate stress, and at prolonged stress were compiled based on the individual egg sampled (*n* = 810 data points: 30 eggs × 9 pens × 3 time collections). The values obtained for shell deformation and shell thickness were log_10_ transformed to improve normality. Percent shell reflectivity data were converted to proportions and logit transformed, and yolk color score data were square-root-transformed. General linear mixed models were fitted, with stressor time point and rearing treatment as fixed effects including their interaction, and pen nested within rearing treatments as a random effect.

## Results

### Welfare Assessment

**Figure 1:**
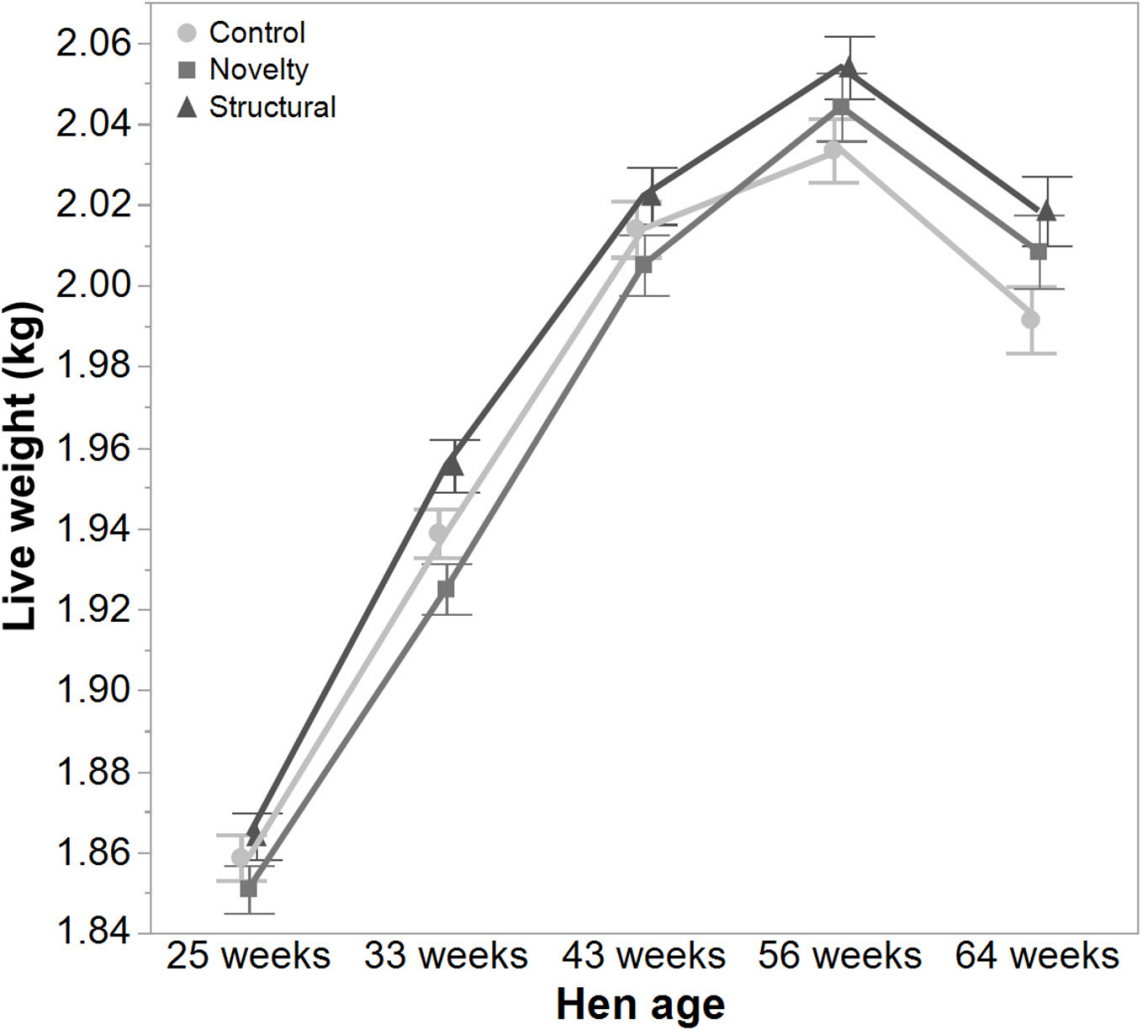
The mean ± SEM of live weight (kg) of hens from different rearing treatments (control, novelty, structural) at different age points (25, 33, 43, 56, 64 weeks) in their laying cycle. The rearing treatments and age of hen interacted significantly (*P* < 0.003).

**Figure 2:**
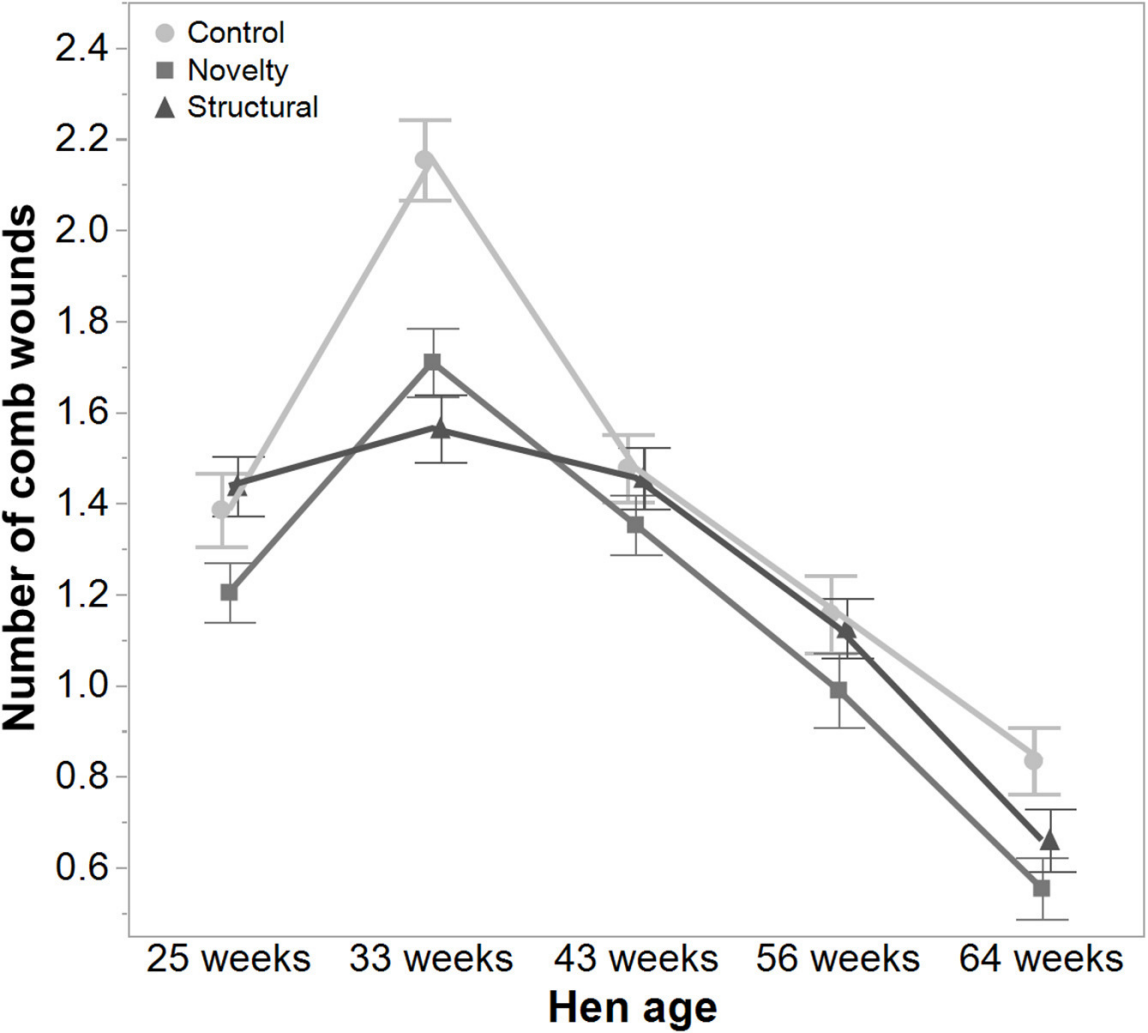
The mean ± SEM of the number of comb wounds in hens from different rearing treatments (control, novelty, structural) at different age points (25, 33, 43, 56, 64 weeks) in their laying cycle. The rearing treatments and age of hen interacted significantly (*P* < 0.0001). Raw data are presented with analysis conducted on transformed data.

**Figure 3:**
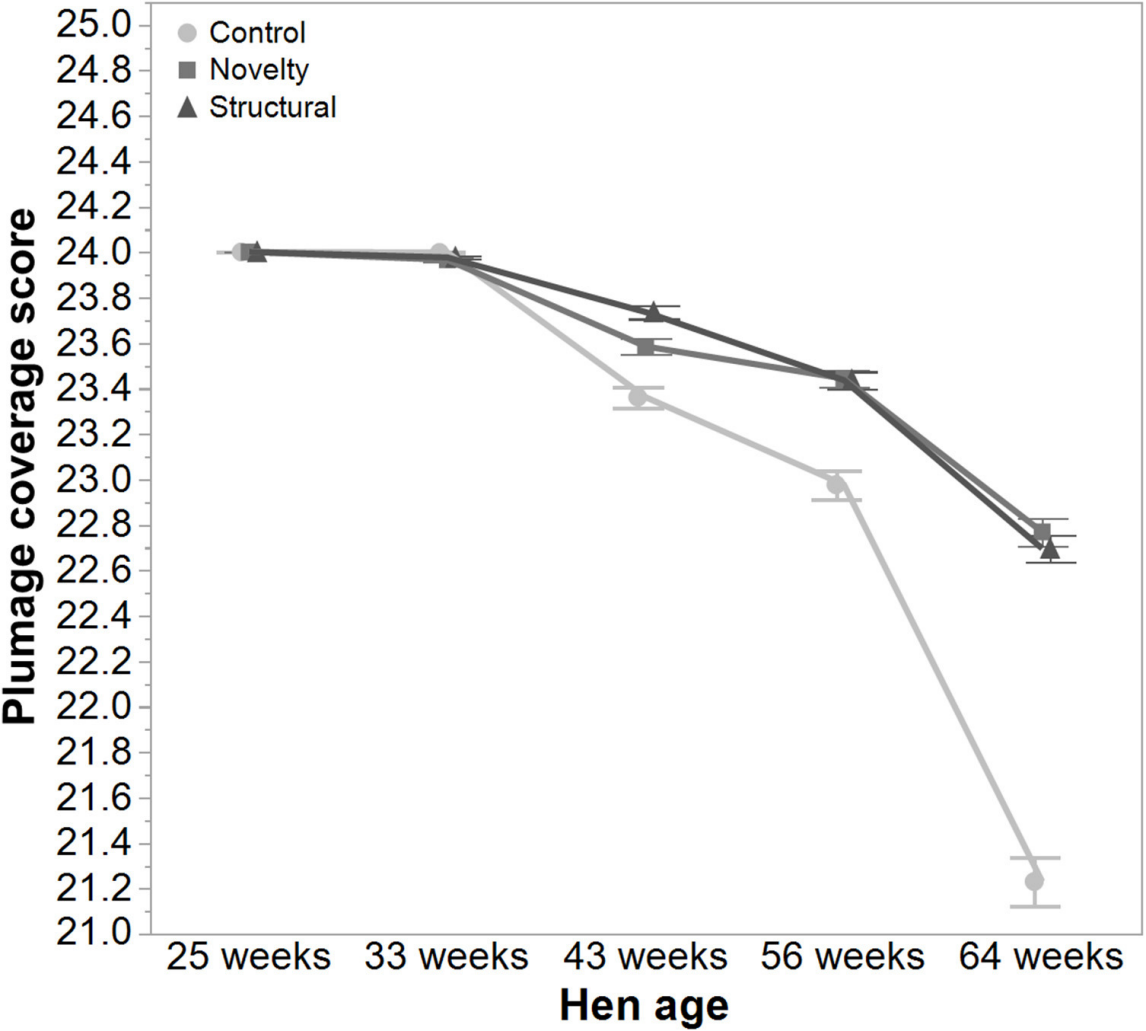
The mean ± SEM of plumage score of hens from different rearing treatments (control, novelty, structural) at different age points (25, 33, 43, 56, 64 weeks) in their laying cycle. The rearing treatments and age of hen interacted significantly (*P* < 0.0001). Lower scores reflect poorer plumage condition. Raw data are presented with the analysis conducted on transformed data.

**Figure 4:**
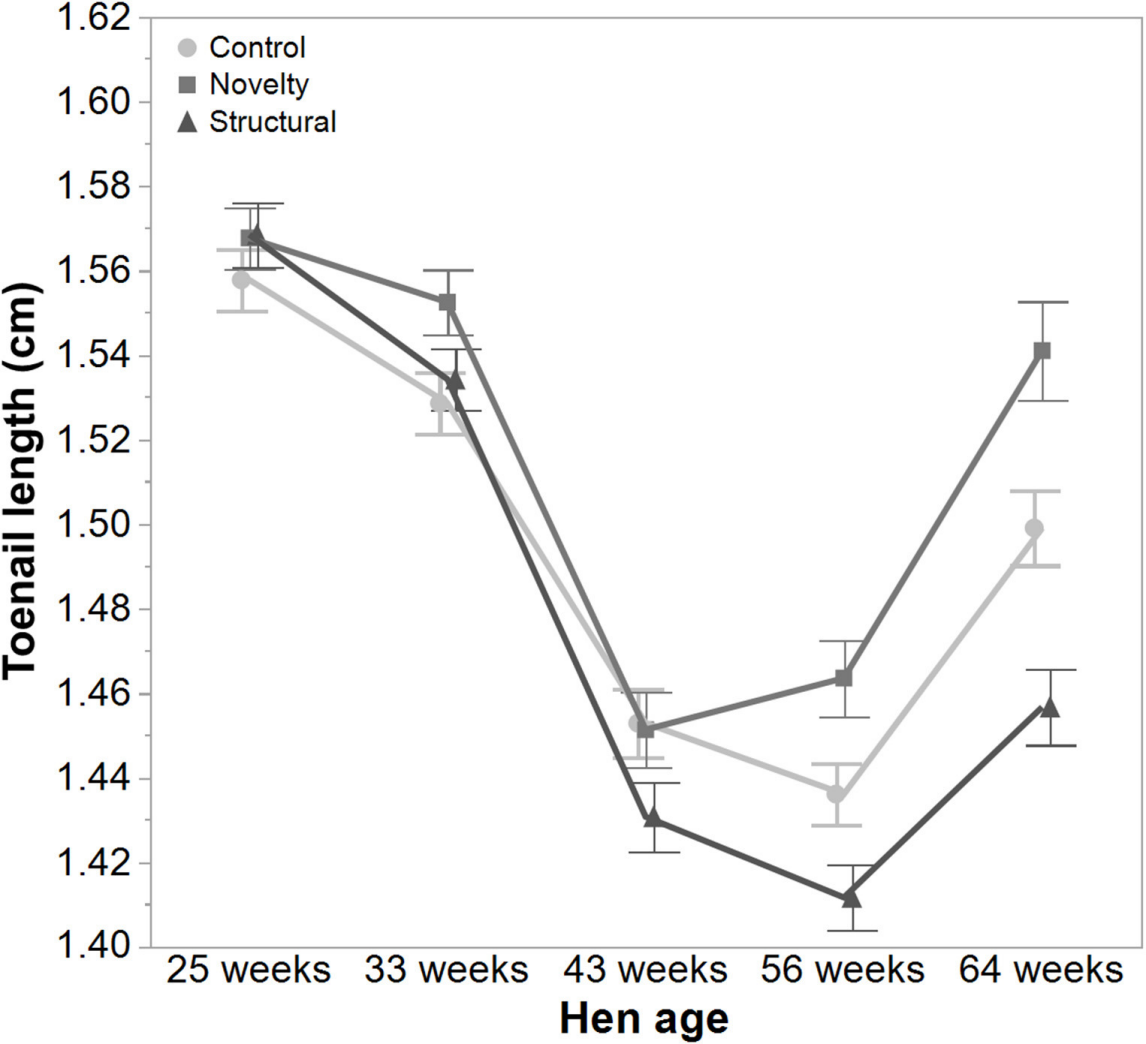
The mean ± SEM of toenail length (cm) of hens from different rearing treatments (control, novelty, structural) at different age points (25, 33, 43, 56, 64 weeks) in their laying cycle. The rearing treatments and age of hen interacted significantly (*P* < 0.0001). Raw data are presented with analysis conducted on transformed data.

There was a significant interaction between hen age and rearing treatments for the live weight of free-range hens [*F*_(8, 5475)_ = 5.46, *P* < 0.003] where the control hens showed a smaller increase in body weight at 56 weeks of age and a greater reduction at 64 weeks of age compared to the structural and novelty hens (Figure 1). However, all hens showed similar trends across age with increases in live weight up until 56 weeks followed by a decrease at 64 weeks of age (Figure 1). There was also a significant interaction between hen age and rearing treatments for the average number of comb wounds [*F*_(8, 5490)_ = 5.47, *P* < 0.0001] with the control hens showing a greater increase in wound numbers at 33 weeks of age (Figure 2). Similar patterns of change were observed across age for all hens with an increase at 33 weeks followed by a decrease across the flock cycle (Figure 2). There was a significant interaction between hen age and rearing treatments for plumage coverage [*F*_(8, 5476)_ = 86.43, *P* < 0.0001], where both the novelty and structural hens had better plumage coverage than the control hens at the later ages (Figure 3). All hens did show a reduction in plumage coverage across age from 43 weeks onwards (Figure 3). There was a significant interaction between hen age and rearing treatments for toenail length [*F*_(8, 5482)_ = 11.30, *P* < 0.0001] where the structural hens had the shortest toenails at the later ages, and the novelty hens the longest (Figure 4). All hens showed similar changes across age with an initial decrease in toenail length followed by an increase at 56 and 64 weeks of age (Figure 4).

An ordinal logistic regression showed that both the age of hen (mean score ± SEM: 33 weeks 0.23 ± 0.01, 43 weeks 0.14 ± 0.01, 56 weeks 0.12 ± 0.01, 64 weeks 0.10 ± 0.01) (χ^2^ = 104.07, df = 4, *P* < 0.0001) and rearing treatment (mean score ± SEM: control 0.15 ± 0.01, novelty 0.08 ± 0.01, structural 0.12 ± 0.01) (χ^2^ = 31.05, df = 2, *P* < 0.0001) had a significant relationship with the beak score of free-range hens, but no significant interaction (*P* = 0.10), so this was removed from the final model. Both the age of hens (mean score ± SEM: 25 weeks: 1.03 ± 0.004, 33 weeks 1.01 ± 0.002, 43 weeks 1.05 ± 0.01, 56 weeks 1.09 ± 0.01, 64 weeks 1.09 ± 0.01) (χ^2^ = 142.65, df = 4, *P* < 0.0001) and rearing treatment (mean score ± SEM: control 1.05 ± 0.001, novelty 1.05 ± 0.001, structural 1.07 ± 0.001) (χ^2^ = 13.43, df = 2, *P* = 0.001) had a significant relationship with the keel score of free-range hens but no significant interaction (*P* = 0.18), so this was removed from the final model. The age of hens had a significant relationship (χ^2^ = 172.92, df = 4, *P* < 0.0001) with the footpad score (mean score ± SEM: 25 weeks: 4 ± 0, 33 weeks 3.96 ± 0.005, 43 weeks 3.98 ± 0.005, 56 weeks 3.99 ± 0.004, 64 weeks 3.93 ± 0.008), but rearing treatment did not (χ^2^ = 4.21, df = 4, *P* = 0.12) and there was no significant interaction (*P* = 0.16), so this was removed from the final model. Overall, across age, the beak score decreased (better beak condition), the footpad scores decreased (worse footpad condition), and the keel scores increased (worse keel condition). The structural group had higher keel scores, and the novelty group had lower beak scores than the other groups. Across the study period, few other health issues were observed, and most occurred when the hens were older. In total, the documented health issues comprised: control group: one hen observed wheezing, seven with prolapses, three with enlarged crops, and three with swollen abdomens; novelty group: six hens with prolapses; structural group: eight hens with prolapses, one with an enlarged crop, and four with swollen abdomens.

### Relationship Between Hen Welfare and Ranging

The test statistics for relationships between welfare parameters and ranging are shown in Table 1. There was a significant negative relationship between live weight and ranging at 56 and 64 weeks of age (both *P* < 0.0001). The beak and keel damage score of hens had significant negative and positive relationships with outdoor ranging at 43 (both *P* = 0.04) and 64 (*P* = 0.0003 and 0.0004) weeks of age, respectively. Footpad scores had significant negative relationships with outdoor ranging at 33 and 64 weeks of age (both *P* ≤ 0.002), and a significant positive relationship at 43 weeks (*P* = 0.03). The number of comb wounds and ranging showed a significant negative relationship at 33 (*P* = 0.0005), 56 (*P* = 0.006) and 64 (*P* < 0.0001) weeks of age. The plumage coverage score and ranging had a significant positive relationship at 43, 56, and 64 weeks of age (all *P* < 0.0001). The toenail length of hens and ranging were significantly negatively correlated at all age points including 33, 43, 56 and 64 weeks of age (all *P* < 0.0001). Overall, ranging affected several welfare parameters but the strongest relationship (*R*^2^ value) was between ranging and toenail length (Table 1).

**Table 1:** The regression analyses of welfare parameters with outdoor ranging time (hours per day) of free-range hens at different age points across the flock cycle.

**Parameters**	**Hen age**	r^*^	***R*^**2**^**	***F*- stats**	***P***
Live weight	33 weeks	−0.02	0.0006	*F*_(1, 1381)_ = 0.82	0.36
	43 weeks	−0.04	0.002	*F*_(1, 1373)_ = 2.21	0.14
	56 weeks	−0.11	0.01	*F*_(1, 1374)_ = 15.44	<0.0001
	64 weeks	−0.11	0.01	*F*_(1, 1356)_ = 17.10	<0.0001
^a^Beak score	33 weeks	−0.04	0.0009	df = 1, χ^2^ = 1.48	0.22
	43 weeks	−0.05	0.004	df = 1, χ^2^ = 4.29	0.04
	56 weeks	−0.13	0.02	df = 1, χ^2^ = 22.12	<0.0001
	64 weeks	−0.10	0.02	df = 1, χ^2^ = 13.31	0.0003
^a^Keel score	33 weeks	−0.002	0.0001	df = 1, χ^2^ = 0.008	0.93
	43 weeks	0.05	0.007	df = 1, χ^2^ = 4.05	0.04
	56 weeks	0.05	0.003	df = 1, χ^2^ = 2.75	0.10
	64 weeks	0.10	0.01	df = 1, χ^2^ = 12.33	0.0004
^a^Footpad score	33 weeks	−0.06	0.02	df = 1, χ^2^ = 7.66	0.006
	43 weeks	0.06	0.02	df = 1, χ^2^ = 5.23	0.02
	56 weeks	−0.009	0.004	df = 1, χ^2^ = 0.07	0.79
	64 weeks	−0.10	0.02	df = 1, χ^2^ = 13.75	0.0002
Number of comb wounds	33 weeks	−0.11	0.009	*F*_(1, 1381)_ = 12.17	0.0005
	43 weeks	−0.007	0.0002	*F*_(1, 1373)_ = 0.23	0.64
	56 weeks	−0.08	0.005	*F*_(1, 1374)_ = 7.57	0.006
	64 weeks	−0.08	0.005	*F*_(1, 1356)_ = 6.62	0.01
Plumage score	33 weeks	0.04	0.002	*F*_(1, 1381)_ = 2.14	0.14
	43 weeks	0.17	0.03	*F*_(1, 1373)_ = 42.14	<0.0001
	56 weeks	0.25	0.06	*F*_(1, 1374)_ = 91.74	<0.0001
	64 weeks	0.22	0.05	*F*_(1, 1356)_ = 71.46	<0.0001
Toenail length	33 weeks	−0.18	0.03	*F*_(1, 1381)_ = 43.57	<0.0001
	43 weeks	−0.39	0.15	*F*_(1, 1373)_ = 237.92	<0.0001
	56 weeks	−0.43	0.18	*F*_(1, 1374)_ = 304.21	<0.0001
	64 weeks	−0.49	0.23	*F*_(1, 1356)_ = 407.50	<0.0001

a *Subjected to ordinal logistic regression and Spearman's correlation*.

* *A correlation coefficient is included to display the direction of the relationship*.

### Stressor and Rearing Treatment Effects on Ranging Behavior and Albumen Corticosterone

**Figure 5:**
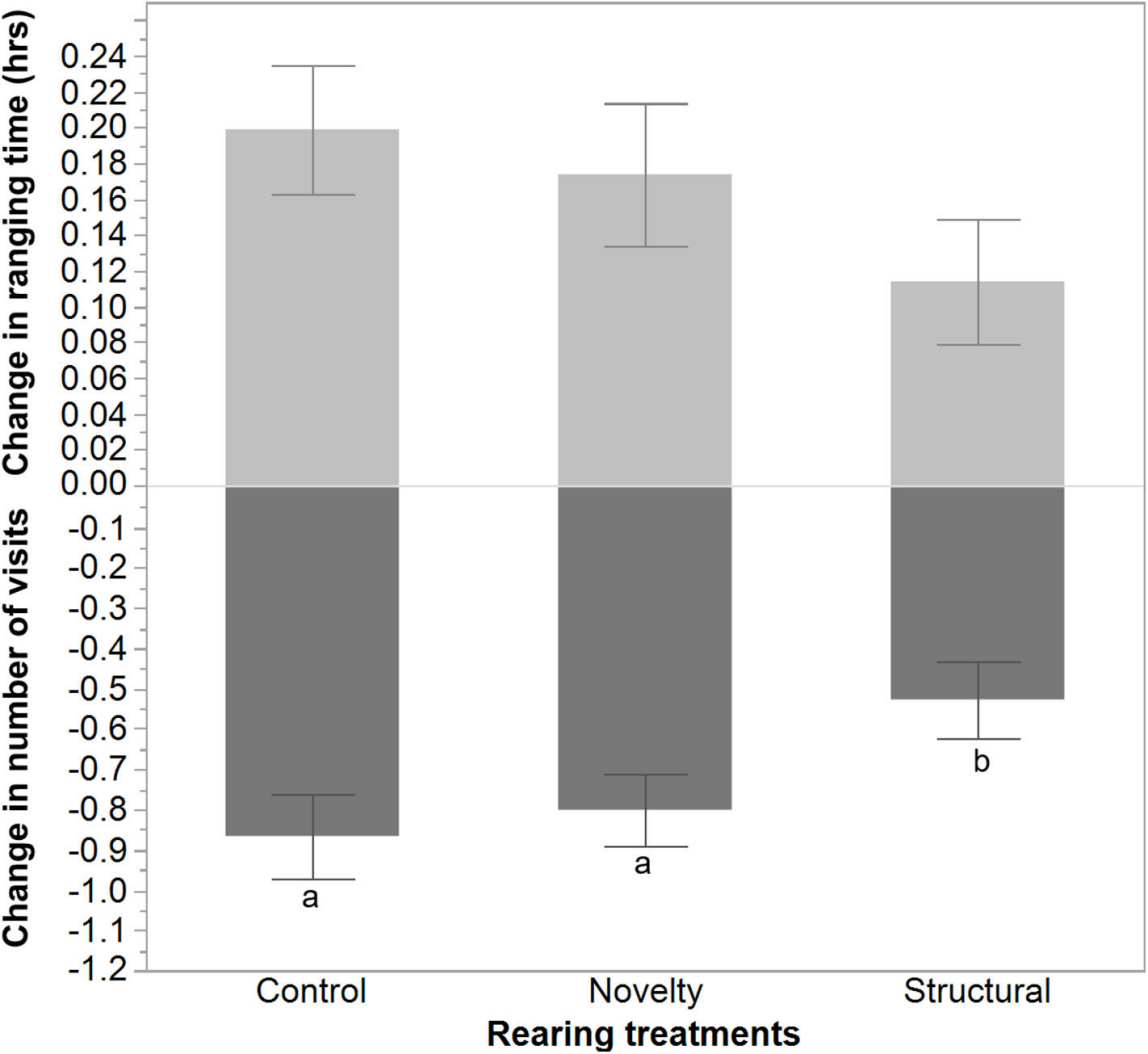
The mean ± SEM of the changes in ranging time (hours) and number of visits due to the applied stress on free-range hens from different rearing treatments (control, novelty, structural). ^a,b^Dissimilar superscript letters indicate significant differences between the change in the number of visits across different rearing treatments (*P* < 0.05).

The average number of visits outside increased following range area shrinkage and varied between rearing treatments with a lower increase in the number of visits for the structural group hens [*F*_(2, 1300)_ = 3.51, *P* = 0.03, Figure 5]. In contrast, the hens' ranging time (h) decreased but did not differ significantly between the rearing treatments [*F*_(2, 1300)_ = 1.41, *P* = 0.24].

**Figure 6:**
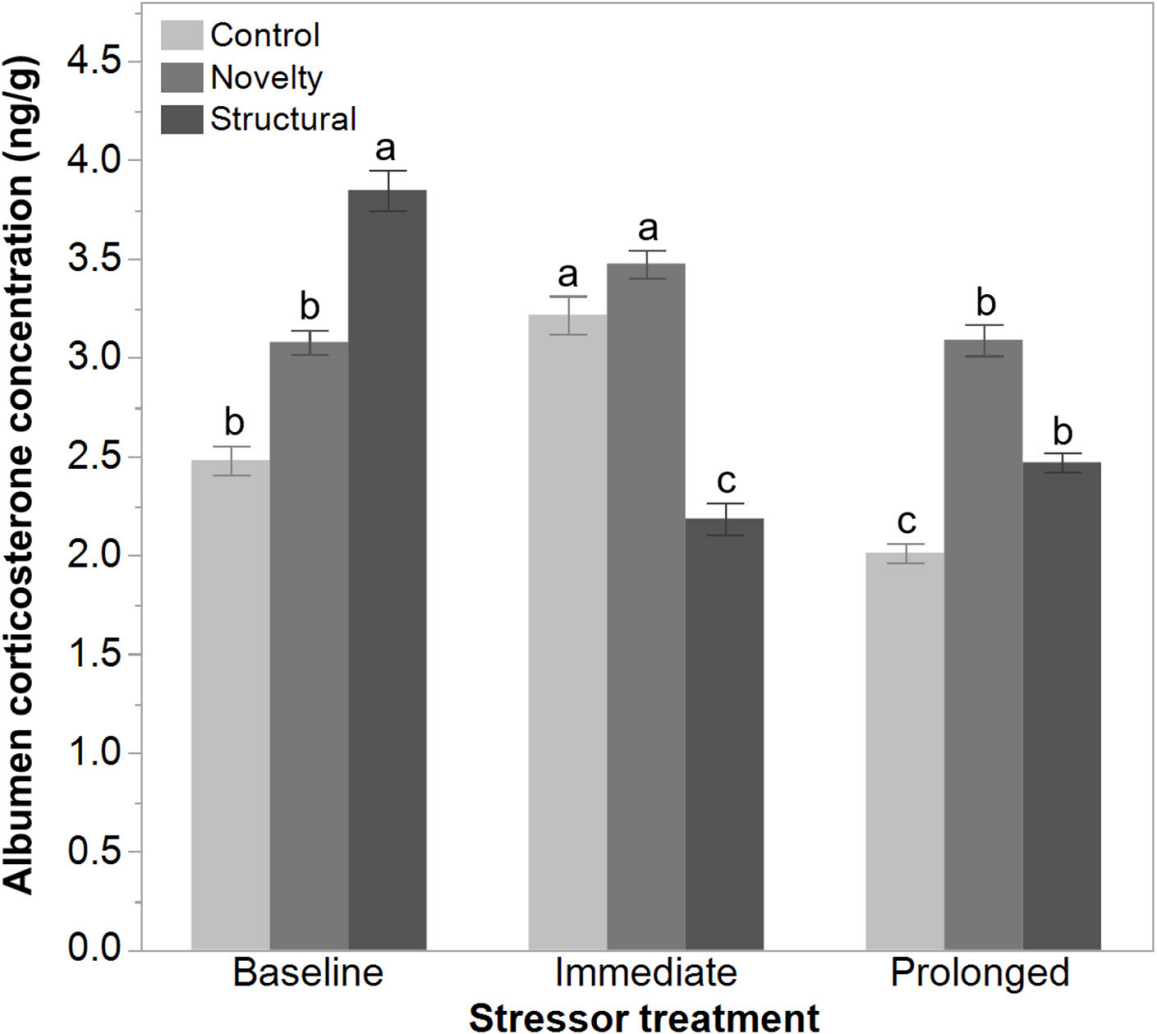
The albumen corticosterone concentrations (ng/g) of eggs from 43 to 45-week old free-range hens from different rearing treatments (control, novelty, structural) across an implemented stressor period (baseline, immediate, prolonged). The interaction between rearing and stressor treatments was significant (*P* < 0.006) but ^a,b,c^dissimilar superscript letters indicate the significant difference across stressor time only.

There was a significant interaction between rearing treatments and the imposed stressor treatment on the egg albumen corticosterone concentrations at 43 weeks of age [*F*_(4, 1314)_ = 90.29, *P* < 0.006]. The corticosterone concentration of both the control and novelty group of hens increased immediately following the range shrinkage but then decreased at the prolonged stress time point. In contrast, the albumen corticosterone concentration in structural hens decreased immediately following the range shrinkage and then increased slightly at the prolonged stress time point (Figure 6).

### Stressor and Rearing Treatment Effects on Egg Quality

The rearing treatments did not significantly affect the egg quality parameters (all *P* ≥ 0.30) but the stressor treatment did (Table 2). The eggshell reflectivity (*P* < 0.0001), egg weight (a trend at *P* = 0.06), breaking strength (*P* = 0.004), shell deformation (*P* = 0.0008), and shell weight (*P* = 0.02) all decreased as the stressor time increased. In contrast, the albumen height and Haugh unit increased across the stressor duration (both *P* < 0.0001). The yolk color score fluctuated (*P* = 0.0002) with an increase in the immediate stress period indicating darker yolks and then a decrease at the prolonged stress period corresponding to a lighter colored yolk (Table 2). There were no significant interactions for any of the parameters (*P* ≥ 0.09) except for eggshell weight [*F*_(4, 795)_ = 2.63, *P* < 0.006]. In the immediate and the prolonged stress period, the eggshell weight of the structural group showed an increase followed by a decrease in shell weight, whereas the opposite pattern was seen for the other treatment groups.

**Table 2:** The least squares means (LSM) ± standard error of the mean (SEM) of egg quality parameters across the implemented stressor period (baseline, immediate, prolonged).

**Parameters**	**Stress treatment**			**SEM**	***F*, df**	***P***
	**Baseline**	**Immediate**	**Prolonged**			
Shell reflectivity	30.75^a^	26.30^b^	26.71^b^	0.25	*F*_(2, 799)_ = 96.08	<0.0001
Egg weight (g)	62.69^a^	61.87^b^	61.97^a^^b^	0.35	*F*_(2, 799)_ = 2.86	0.06
Albumen height (mm)	9.96^b^	10.36^a^	10.55^a^	0.13	*F*_(2, 799)_ = 13.61	<0.0001
Haugh unit	98.10^b^	100.30^a^	101.00^a^	0.60	*F*_(2, 799)_ = 16.05	<0.0001
Yolk color score	10.34^c^	10.65^a^	10.50^b^	0.09	*F*_(2, 799)_ = 8.52	0.0002
Breaking strength (N)	46.85^a^	45.10^b^	45.06^b^	0.53	*F*_(2, 799)_ = 5.48	0.004
Shell deformation (mm)	0.29^a^	0.28^b^	0.27^b^	0.004	F_(2, 799)_ = 7.23	0.0008
Shell weight (g)	6.13^a^	6.03^b^	6.04^b^	0.03	*F*_(2, 795)_ = 3.99	0.02
Shell thickness (mm)	0.43	0.43	0.43	0.002	*F*_(2, 799)_ = 0.61	0.54

a−c *Means with different superscript letters in each row differ significantly (P < 0.05). Raw data are presented in the table with some analyses conducted on transformed data*.

## Discussion

We evaluated the impacts of rearing enrichments on, and associations of, outdoor ranging with welfare parameters of free-range hens across the laying cycle along with their adaptation to an imposed environmental stressor. Rearing treatments affected welfare parameters of plumage coverage, toenail length, and body weight with greater differences seen between treatments as the hens aged. Typically both types of enriched hens were different from the control hens and showed improved welfare but not exclusively across all measured parameters. The structural hens showed more keel bone damage. Results on ranging patterns from the same flock of hens showed that the structural hens spent more time outside and the novelty hens had fewer visits to the range; both enriched groups had longer individual visits than the control hens (32). Welfare parameters of body weight, comb wounds, toenail length, beak damage and footpad condition decreased with range use, and keel bone damage increased but inconsistently across the measured age points. Plumage coverage improved with range use across most age points. The average number of visits outside increased due to the imposed stressor and varied between rearing treatments with a lower increase in the number of visits in the structural group of hens. Correspondingly, the structural hens showed contrasting changes in albumen corticosterone concentrations where the corticosterone decreased immediately after the implementation of the stressor but increased in the control and novelty hens. There were clear impacts of the stressor treatment on all egg quality parameters. The limitation of only three replicates per treatment due to the confounds of the available experimental facilities must be acknowledged in the interpretation of the findings.

Enriched hens had better plumage coverage throughout the laying cycle. A subset of hens with the most extreme ranging patterns (nil, low, and high range use) from the same flock as the current study also showed better plumage in the enriched hens than the non-enriched hens at the later stage of the laying cycle (16). Plumage losses are typically the result of the feather pecking behavior of hens. Rearing enrichments might affect the development of pullets' behavior (33) such as increasing exploratory behavior (20, 34) and navigation abilities (20), subsequently affecting their movement both indoors and outdoors (19). Hens that spend more time exploring and foraging outside may consequently reduce the time spent feather pecking conspecifics or be better able to avoid being pecked. As the hens from different rearing treatments also showed differences in ranging behavior (32), it is unclear whether the effects of the rearing treatments were related to behavioral differences that developed during the rearing period, if they were a consequence of the variation in range use, or a combination of both. Reductions in feather pecking behavior and/or improvements in plumage have previously been demonstrated to be associated with greater use of the range area (9, 10, 35), although Larsen et al. (15) found no association between plumage condition and individual outdoor ranging. Feather pecking might also be associated with negative affective states such as fear (36) which could be mitigated by increased exercise, a hypothesis that warrants further investigation in ranging hens. Differences in pecking behavior may be related to differences in social interactions. Early feather pecking behavior is evidenced to be associated with social exploration (37), and in a previous study with free-range hens, there were differences in synchronized group-level ranging patterns between enriched/non-enriched hens (38). In support of this, the most comb wounds were seen at 33 weeks of age and more so in the control hens than both enriched groups. This might be a result of the social restructuring in the group when they started to use the range. Pop-holes were first opened at 25 weeks of age, but range use was initially low (32). At 33 weeks of age when range use was increasing, the indoor stocking density lowered and potentially resulted in the reorganization of social hierarchies. The control hens with the most comb wounds may have been poorer at managing their social interactions.

Other welfare parameters were also associated with range use with more differences seen as the hens aged. Similarly, hours spent outside increased as the hens aged followed by a drop from 56 to 64 weeks of age which was likely affected by the summer season (32). However, although there were significant relationships for keel damage, beak condition, footpad condition, comb wound count, and body weight, the *R*^2^ values were very low indicating that the ranging patterns only accounted for some of the variability that was seen. Keel bone damage was higher in the structural hens, but they also showed more ranging (32), which may have contributed to this difference between the rearing treatments. Larsen et al. (15) also found few associations between welfare indicators and range use variability in commercial free-range hens. These authors hypothesized that the choice provided in the free-range environment allows each hen to range to the degree that meets their own needs; thus, natural individual variation in ranging may not have detectable implications for welfare. The impacts of range use may, however, be stronger for hens that show more extreme ranging patterns as were selected in a subsample of hens from the current study (16). Additionally, forced ranging patterns disparate from natural choices (i.e., reducing range access hours, or forcing hens outside) may have greater effects on individual welfare, but this hypothesis remains to be tested. The clearest relationship between ranging and welfare scoring was the reduction of toenail length for hens that spent more time outside. This result has previously been demonstrated (13, 17), and it is expected that more time walking/scratching in the dirt would maintain suitable toenail lengths which can reduce the risks of getting toenails caught in the structure of the system.

The applied environmental stressor impacted the hens' ranging by decreasing the ranging time outdoors while increasing the number of visits outside. There was no effect of rearing treatment on the change in time spent outside, but the structural hens showed a lower increase in the number of outdoor visits. These ranging behavior results are similar to those of a previous study conducted in the same facility that applied the same stressor to smaller flocks of hens exposed to enrichments (or not) for the first 3 weeks of life (19). However, in this previous study, the enriched hens (visual, auditory, structural enrichments) showed a greater increase in the number of visits relative to non-enriched hens (19). Physiologically, the structural hens actually showed a decrease in albumen corticosterone when sampled 3 days after the range area was reduced, compared with an increase in the control and novelty hens. This result is similar to the corticosterone responses following the first week of range access in the same flock of hens where the structural hens showed the smallest increase compared with the other treatment groups and had higher baseline levels (32). In the previous study the enriched hens also showed lower increases in corticosterone following imposed stressors (19). The structural hens in the current study may have been more adaptable to environmental change showing comparatively lower behavioral modification and a lower physiological stress response; however, the mechanism for this is unclear. The lower physiological response and comparatively lower behavioral response does not align with coping styles, as in both active and passive responses, the direction of change between behavioral and physiological parameters oppose each other (39). The structural hens may have developed improved adaptability through the perching structures during rearing that included both height and opaque panels. This may have enabled the pullets to exhibit avoidance behaviors as needed (e.g., perching as a predator avoidance strategy) which stimulated coping. Further research would be needed to explore this idea. The higher degree of outdoor ranging prior to implementation of the stressor may have also meant these hens were getting more exercise, which modified the functioning of their hypothalamic pituitary adrenal axis and advanced their rate of physiological adaptation to the stressor (40). The complex relationship between baseline metabolism and glucocorticoids may have been impacted by typical ranging differences between the treatment groups (41, 42). All hens reduced ranging time, but the control and novelty hens increased their visits, whereas the structural hens did not to the same degree; thus, their overall ranging activity was lower during the stressor period. The validated radioimmunoassay to determine the corticosterone concentrations used antiserum that does have some cross-reactivities to other steroids (31), so it is unclear to what degree these may have affected the results. Blood profiles in future testing could be more informative or provide additional measures (41) but require invasive sampling techniques. Further studies could also measure the rate of adaptation following the removal of an imposed stressor, which was not assessed in this study, including measuring the use of the available range area rather than just time and visits outside.

It is possible that the structural enrichments during rearing resulted in neurological changes such as greater hemispheric flexibility that improved adaptive responses to their environments (43). Previous comparisons between cage-reared and aviary-reared hens showed functional lateralization in the hippocampus and caudolateral nidopallium but no differences between the rearing treatments, although all birds were in similar environments for the first 4 weeks of rearing (44). Campbell et al. (45) found no differences in the telencephalon or hippocampal volume between enriched-reared and non-enriched-reared hens. However, multiple studies in rodents have demonstrated increased synaptic plasticity in the hippocampus of animals exposed to enrichments, particularly short-term (46). The impacts of rearing on neural maturation warrant further investigation.

Environmental stressors did affect egg quality with eggshell reflectivity, egg weight, breaking strength, shell deformation, and shell weight showing decreases, but albumen height and Haugh units showed increases across the stressor period. However, distinct from the rearing treatment effects in behavioral and corticosterone change, the effects on egg quality were similar across all groups of hens. Some of these effects of stress on egg parameters were similar to impacts of heat stress and disease on egg quality (27, 47, 48). Decreases in yolk color also correspond with the effects of dietary corticosterone but the increased albumen height is opposite to previous reports of corticosterone supplementation (26) or heat stress (49). The implemented stressor and changes in corticosterone may have affected albumen proteins (50), but changes in activity levels (ranging behavior) and potentially feed intake may have also had impacts on nutrient allocation as rearing treatment differentially affected corticosterone concentrations but not egg quality parameters. The egg weight and shell characteristics including eggshell color were decreased due to stress, which might be related to reduced feed intake, particularly calcium which could have affected breaking strength, shell deformation, and shell weight. However, feed intake was not measured in this study, and thus, further research is warranted to clarify this.

## Conclusion

Overall, enrichments in rearing provided welfare benefits at some age points, including better plumage coverage, fewer comb wounds, and shorter toenails, but this was likely associated with the differences in ranging also seen between the rearing treatment groups. Ranging was related to primarily improved welfare parameters of free-range hens, but these relationships had high individual variability. Structural enrichments may have improved adaptation by minimizing both behavioral changes and immediate physiological stress responses. Change in resource access decreased egg quality, but rearing enrichments did not minimize these effects. Rearing enrichments along with optimum range access could be recommended for positive effects on hen welfare. However, this study only had three replicates per treatment due to the limitations of the experimental facilities, and thus, longitudinal studies with increased replicates and in commercial settings to clarify the relationship between individual range use and welfare parameters are warranted.

## Data Availability Statement

The raw data supporting the conclusions of this article will be made available by the authors, without undue reservation.

## Ethics Statement

The animal study was reviewed and approved by University of New England Animal Ethics Committee.

## Author Contributions

DC conceived and designed the experiment. MB and DC collected and analyzed the data, and prepared the figures and tables. MB drafted the manuscript. JD performed the corticosterone analyses. TD and CL assisted in data collection, experimentation, and project administration. MB and DC revised the manuscript, all authors approved the final version, and contributed significantly to this manuscript.

## Conflict of Interest

The authors declare that the research was conducted in the absence of any commercial or financial relationships that could be construed as a potential conflict of interest.

## References

[1] Edge M Barnett J Development of animal welfare standards for the livestock transport industry: process, challenges, and implementation. J Vet Behav Clin Appl Res. (2009) 4:187–92. 10.1016/j.jveb.2009.07.001

[2] Vanhonacker F Van Poucke E Tuyttens F Verbeke W Citizens' views on farm animal welfare and related information provision: exploratory insights from Flanders, Belgium. J Agric Environ Ethics. (2010) 23:551–69. 10.1007/s10806-010-9235-9

[3] Pettersson IC Weeks CA Wilson LRM Nicol CJ Consumer perceptions of free-range laying hen welfare. Br Food J. (2016) 118:1999–2013. 10.1108/BFJ-02-2016-0065

[4] Moffat K Murphy S Boughen N Australian Egg Industry Community Research Report 2019. CSIRO (2020).

[5] Heng Y Peterson HH Li X Consumer attitudes toward farm-animal welfare: the case of laying hens. J Agr Resour Econ. (2013) 38:418–34. 10.22004/ag.econ.165936

[6] Bray HJ Ankeny RA Happy chickens lay tastier eggs: motivations for buying free-range eggs in Australia. Anthrozoös. (2017) 30:213–26. 10.1080/08927936.2017.1310986

[7] Bennett RM Jones PJ Nicol CJ Tranter RB Weeks CA Consumer attitudes to injurious pecking in free range egg production. Anim Welf. (2016) 25:91–100. 10.7120/09627286.25.1.091

[8] Campbell DLM Bari MS Rault J-L. Free-range egg production: its implications for hen welfare. Anim Prod Sci. (2020). 10.1071/AN19576. [Epub ahead of print].27328829

[9] Chielo LI Pike T Cooper J. Ranging behaviour of commercial free-range laying hens. Animals. (2016) 6:1–13. 10.3390/ani6050028 27128946 PMC4880845

[10] Rodriguez-Aurrekoetxea A Estevez I. Use of space and its impact on the welfare of laying hens in a commercial free-range system. Poult Sci. (2016) 95:2503–13. 10.3382/ps/pew238 27433014

[11] Pettersson IC Weeks CA Nicol CJ Provision of a resource package reduces feather pecking and improves ranging distribution on free-range layer farms. Appl Anim Behav Sci. (2017) 195:60–66. 10.1016/j.applanim.2017.06.007

[12] Lambton SL Knowles TG Yorke C Nicol CJ. The risk factors affecting the development of gentle and severe feather pecking in loose housed laying hens. Appl Anim Behav Sci. (2010) 123:32–42. 10.1016/j.applanim.2009.12.01023603726

[13] Yilmaz Dikmen B Ipek A Sahan Ü Petek M Sözcü A. Egg production and welfare of laying hens kept in different housing systems (conventional, enriched cage, and free range). Poult Sci. (2016) 95:1564–72. 10.3382/ps/pew082 26994200

[14] Campbell DLM Hinch GN Downing JA Lee C Outdoor stocking density in free-range laying hens: effects on behaviour and welfare. Animal. (2017) 11:1036–45. 10.1017/S1751731116002342 27821220

[15] Larsen H Hemsworth PH Cronin GM Gebhardt-Henrich SG Smith CL Rault J-L. Relationship between welfare and individual ranging behavior in commercial free-range laying hens. Animal. (2018) 12:2356–64. 10.1017/S1751731118000022 29362002

[16] Bari MS Laurenson YCSM Cohen-Barnhouse AM Walkden-Brown SW Campbell DLM. Effects of outdoor ranging on external and internal health parameters for hens from different rearing enrichments. PeerJ. (2020) 8:e8720. 10.7717/peerj.8720 32185113 PMC7061908

[17] Campbell DLM Hinch GN Dyall TR Warin L Little BA Lee C. Outdoor stocking density in free-range laying hens: radio-frequency identification of impacts on range use. Animal. (2017) 11:121–30. 10.1017/S1751731116001154 27328829

[18] Janczak AM Riber AB. Review of rearing-related factors affecting the welfare of laying hens. Poult Sci. (2015) 94:1454–69. 10.3382/ps/pev123 26009752 PMC4991062

[19] Campbell DLM Hinch GN Downing JA Lee C. Early enrichment in free-range laying hens: effects on ranging behaviour, welfare and response to stressors. Animal. (2018) 12:575–84. 10.1017/S1751731117001859 28756797

[20] Gunnarsson S Yngvesson J Keeling LJ Forkman B. Rearing without early access to perches impairs the spatial skills of laying hens. Appl Anim Behav Sci. (2000) 67:217–28. 10.1016/S0168-1591(99)00125-2 10736530

[21] Moe RO Guemene D Bakken M Larsen HJS Shini S Lervik S. Effects of housing conditions during the rearing and laying period on adrenal reactivity, immune response and heterophil to lymphocyte (H/L) ratios in laying hens. Animal. (2010) 4:1709–15. 10.1017/S175173111000100X 22445125

[22] Vestergaard KS Skadhauge E Lawson L. The stress of not being able to perform dustbathing in laying hens. Physiol Behav. (1997) 62:413–19. 10.1016/S0031-9384(97)00041-3 9251988

[23] Mirfendereski E Jahanian R. Effects of dietary organic chromium and vitamin C supplementation on performance, immune responses, blood metabolites, and stress status of laying hens subjected to high stocking density. Poult Sci. (2015) 94:281–88. 10.3382/ps/peu074 25650433

[24] Carvalho RR Palme R da Silva Vasconcellos A. An integrated analysis of social stress in laying hens: the interaction between physiology, behaviour, and hierarchy. Behav Process. (2018) 149:43–51. 10.1016/j.beproc.2018.01.016 29408572

[25] Engel J Widowski T Tilbrook A Butler K Hemsworth P. The effects of floor space and nest box access on the physiology and behavior of caged laying hens. Poult Sci. (2019) 98:533–47. 10.3382/ps/pey378 30165652

[26] Kim Y-H Kim J Yoon H-S Choi Y-H. Effects of dietary corticosterone on yolk colors and eggshell quality in laying hens. Asian-Australas J Anim Sci. (2015) 28:840–46. 10.5713/ajas.14.0849 25925061 PMC4412980

[27] Mertens K Vaesen I Loffel J Kemps B Kamers B Perianu C. The transmission color value: a novel egg quality measure for recording shell color used for monitoring the stress and health status of a brown layer flock. Poult Sci. (2010) 89:609–17. 10.3382/ps.2009-00261 20181881

[28] Primary Industries Standing Committee. Model Code of Practice for the Welfare of Animals: Domestic Poultry. Collingwood, VIC: CSIRO Publishing (2002).

[29] Hy-line Management Guide for Hy-Line Brown Laying Hen in Alternative System. (2016). Available online at: https://www.hyline.com/userdocs/pages/B_ALT_COM_ENG.pdf (accessed October 23, 2017)

[30] Tauson R Kjaer J Maria GA Cepero R Holm KE Applied scoring of integument and health in laying hens. Anim Sci Paper Rep. (2005) 23:153–59.

[31] Downing J Bryden W. Determination of corticosterone concentrations in egg albumen: a non-invasive indicator of stress in laying hens. Physiol Behav. (2008) 95:381–87. 10.1016/j.physbeh.2008.07.001 18657560

[32] Campbell DLM Dyall TR Downing JA Cohen-Barnhouse AM Lee C. Rearing enrichments affected ranging behavior in free-range laying hens. Front Vet Sci. (2020) 7:446. 32923462 10.3389/fvets.2020.00446 PMC7457029

[33] Campbell DLM De Haas EN Lee C. A review of environmental enrichment for laying hens during rearing in relation to their behavioral and physiological development. Poult Sci. (2019) 98:9–28. 10.3382/ps/pey319 30107615 PMC6347129

[34] Krause ET Naguib M Trillmich F Schrader L The effects of short term enrichment on learning in chickens from a laying strain (*Gallus gallus domesticus*). Appl Anim Behav Sci. (2006) 101:318–27. 10.1016/j.applanim.2006.02.005

[35] De Koning C Kitessa SM Barekatain R Drake K Determination of range enrichment for improved hen welfare on commercial fixed-range free-range layer farms. Anim Prod Sci. (2018) 59:1336–48. 10.1071/AN17757

[36] Rodenburg TB Van Krimpen MM De Jong IC De Haas EN Kops MS Riedstra BJ The prevention and control of feather pecking in laying hens: identifying the underlying principles. Worlds Poult Sci J. (2013) 69:361–74. 10.1017/S0043933913000354

[37] Riedstra B Groothuis TG Early feather pecking as a form of social exploration: the effect of group stability on feather pecking and tonic immobility in domestic chicks. Appl Anim Behav Sci. (2002) 77:127–38. 10.1016/S0168-1591(02)00031-X

[38] Campbell DLM Horton BJ Hinch GN. Using radio-frequency identification technology to measure synchronized ranging of free-range laying hens. Animals. (2018) 8:210. 10.3390/ani8110210 30453521 PMC6262442

[39] Cockrem JF Stress, corticosterone responses and avian personalities. J Ornithol. (2007) 148:169–78. 10.1007/s10336-007-0175-8

[40] Hare BD Beierle JA Toufexis DJ Hammack SE Falls WA. Exercise-associated changes in the corticosterone response to acute restraint stress: evidence for increased adrenal sensitivity and reduced corticosterone response duration. Neuropsychopharmacology. (2014) 39:1262–69. 10.1038/npp.2013.329 24280995 PMC3957122

[41] Scanes CG. Biology of stress in poultry with emphasis on glucocorticoids and the heterophil to lymphocyte ratio. Poult Sci. (2015) 95:2208–15. 10.3382/ps/pew137 27252367

[42] McEwen BS Wingfield JC. The concept of allostasis in biology and biomedicine. Horm Behav. (2003) 43:2–15. 10.1016/S0018-506X(02)00024-7 12614627

[43] Rogers LJ Kaplan G Does functional lateralization in birds have any implications for their welfare? Symmetry. (2019) 11:1043 10.3390/sym11081043

[44] Tahamtani FM Nordgreen J Brantsæter M Østby GC Nordquist RE Janczak AM. Does early environmental complexity influence tyrosine hydroxylase in the chicken hippocampus and “prefrontal” caudolateral nidopallium? Front Vet Sci. (2016) 3:8. 10.3389/fvets.2016.00008 26904550 PMC4749677

[45] Campbell DLM Talk AC Loh ZA Dyall TR Lee C. Spatial cognition and range use in free-range laying hens. Animals. (2018) 8:26. 10.3390/ani8020026 29419742 PMC5836034

[46] Ohline SM Abraham WC. Environmental enrichment effects on synaptic and cellular physiology of hippocampal neurons. Neuropharmacology. (2019) 145:3–12. 10.1016/j.neuropharm.2018.04.007 29634984

[47] Lin H Mertens K Kemps B Govaerts T de Ketelaere B de Baerdemaeker J. New approach of testing the effect of heat stress on eggshell quality: mechanical and material properties of eggshell and membrane. Br Poult Sci. (2004) 45:476–82. 10.1080/00071660400001173 15484721

[48] Mashaly MM Hendricks GL Kalama MA Gehad AE Abbas AO Patterson PH. Effect of heat stress on production parameters and immune responses of commercial laying hens. Poult Sci. (2004) 83:889–94. 10.1093/ps/83.6.889 15206614

[49] Seven PT The effects of dietary Turkish propolis and vitamin C on performance, digestibility, egg production and egg quality in laying hens under different environmental temperatures. Asian-Australas J Ani. Sci. (2008) 21:1164–70. 10.5713/ajas.2008.70605

[50] Kim J Choi YH. Differential abundance of egg white proteins in laying hens treated with corticosterone. J Agr Food Chem. (2014) 62:12346–59. 10.1021/jf504469t 25436390

